# A Novel Splice Variant of Human TGF-β Type II Receptor Encodes a Soluble Protein and Its Fc-Tagged Version Prevents Liver Fibrosis *in vivo*

**DOI:** 10.3389/fcell.2021.690397

**Published:** 2021-09-10

**Authors:** Marcela Soledad Bertolio, Anabela La Colla, Alejandra Carrea, Ana Romo, Gabriela Canziani, Stella Maris Echarte, Sabrina Campisano, German Patricio Barletta, Alexander Miguel Monzon, Tania Melina Rodríguez, Andrea Nancy Chisari, Ricardo Alfredo Dewey

**Affiliations:** ^1^Laboratorio de Terapia Génica y Células Madre, Instituto Tecnológico de Chascomús (INTECH), CONICET-UNSAM, Buenos Aires, Argentina; ^2^Departamento de Química y Bioquímica, Facultad de Ciencias Exactas y Naturales, Universidad Nacional de Mar del Plata, Buenos Aires, Argentina; ^3^Drexel U-Sidney Kimmel Cancer Center, Thomas Jefferson U S200 Biosensor Shared Resource, Department of Biochemistry and Molecular Biology, Drexel University College of Medicine, Philadelphia, PA, United States; ^4^Molecular Physics and Biophysics Group, Department of Science and Technology, National University of Quilmes, CONICET, Bernal, Argentina; ^5^Department of Biomedical Sciences, University of Padua, Padua, Italy

**Keywords:** soluble receptor, peptibody, TGF-beta, fusion protein, organ fibrosis

## Abstract

We describe, for the first time, a new splice variant of the human TGF-β type II receptor (TβRII). The new transcript lacks 149 nucleotides, resulting in a frameshift and the emergence of an early stop codon, rendering a truncated mature protein of 57 amino acids. The predicted protein, lacking the transmembrane domain and with a distinctive 13-amino-acid stretch at its C-terminus, was named TβRII-Soluble Endogenous (TβRII-SE). Binding predictions indicate that the novel 13-amino-acid stretch interacts with all three TGF-β cognate ligands and generates a more extensive protein–protein interface than TβRII. TβRII-SE and human IgG1 Fc domain were fused in frame in a lentiviral vector (Lv) for further characterization. With this vector, we transduced 293T cells and purified TβRII-SE/Fc by A/G protein chromatography from conditioned medium. Immunoblotting revealed homogeneous bands of approximately 37 kDa (reduced) and 75 kDa (non-reduced), indicating that TβRII-SE/Fc is secreted as a disulfide-linked homodimer. Moreover, high-affinity binding of TβRII-SE to the three TGF-β isoforms was confirmed by surface plasmon resonance (SPR) analysis. Also, intrahepatic delivery of Lv.TβRII-SE/Fc in a carbon tetrachloride-induced liver fibrosis model revealed amelioration of liver injury and fibrosis. Our results indicate that TβRII-SE is a novel member of the TGF-β signaling pathway with distinctive characteristics. This novel protein offers an alternative for the prevention and treatment of pathologies caused by the overproduction of TGF-β ligands.

## Introduction

Transforming growth factor-β (TGF-β) is a multifunctional cytokine involved in critical processes, including immune regulation and wound healing together with cell proliferation, maturation, and differentiation (Derynck and Budi, 2019). Three TGF-β isoforms have been identified in mammals: TGF-β1, TGF-β2, and TGF-β3. They are encoded by distinct genes sharing 64–82% sequence identity. These genes are regulated developmentally and in a tissue-specific manner (Moses et al., 2016). Although in tissue culture assays all three isoforms display overlapping biological activities, in isoform-specific null mice, their activities are non-overlapping (Shull et al., 1992; Proetzel et al., 1995; Sanford et al., 1997).

Canonical signaling starts when mature, dimeric TGF-β isoforms bind cell surface receptor complexes comprising “type II” (TβRII) and “type I” (TβRI) receptors (Heldin and Moustakas, 2016). TβRI has a short Gly-Ser-rich juxtamembrane sequence (GS domain) that is phosphorylated by TβRII kinase in response to ligand binding (Wieser et al., 1995; Chaikuad and Bullock, 2016; Heldin and Moustakas, 2016). Not all TGF-β ligands contact the receptor complex equally. TGF-β1 and TGF-β3 bind to TβRII receptor dimers tightly without the need for TβRI receptors. On the other hand, TβRI receptors do require TβRII for ligand binding due to its low affinity for TGF-β (Hinck et al., 2016). Conversely, the low affinity of TGF-β2 for TβRII or TβRI receptors suggests that TGF-β2 initially binds, in preformed receptor complexes, with TβRI or TβRII (Ehrlich et al., 2012). Moreover, it is known that the betaglycan coreceptor (former TβRIII), is necessary for efficient binding of TGF-β2 and subsequent signaling (López-Casillas et al., 1993). This coreceptor binds all three TGF-βs showing a comparable affinity for TGF-β2 (Dong et al., 2007). TGF-β2 binds to betaglycan and recruits TβRII and TβRI to phosphorylate TβRI eliciting downstream signaling (López-Casillas et al., 1993).

Ligand binding to the receptor ectodomains induces conformational changes at the ligand–receptor interface, bringing their cytoplasmic domains closer (Chaikuad and Bullock, 2016; Hinck et al., 2016). This stabilization enables TβRII to phosphorylate TβRI at the serine residues of the GS domain, which then induces conformational changes that activate TβRI kinase (Huse et al., 1999; Chaikuad and Bullock, 2016). TβRI activated by TβRII phosphorylates the two C-terminal serines of Smad2 and 3, inducing their activation. These “receptor-activated Smads” or R-Smads then bind Smad4 and translocate into the nucleus, where they activate or repress target genes (Hata and Chen, 2016).

In addition to TβRII protein, the *tgfbr2* gene encodes the membrane-anchor isoform TβRII-B, *via* alternative splicing. This variant involves an insertion of 75 bp coding for 25 amino acids in the extracellular domain, with an isoleucine-to-valine exchange (Hirai and Fijita, 1996). Unlike TβRII, TβRII-B binds and signals directly *via* all three TGF-β isoforms without the requirement of betaglycan (Rotzer et al., 2001). Additionally, in the absence of betaglycan, TGF-β2 binding to TβRII-B requires TβRI (del Re et al., 2004).

The highly diverse and context-dependent TGF-β responses are not supported by the canonical signaling model; therefore, it is an oversimplification (Budi et al., 2017; Derynck and Budi, 2019). Also, TGF-β receptors activate non-Smad signaling pathways contributing to the TGF-β response, including pathways such as PI3K-AKT-mTOR and MAPK (Zhang, 2017), adding more complexity to the signaling cascade. It has become apparent that it is necessary to learn more about the mechanistic involvement of receptor presentation and activation, and the control of cell responsiveness to define the developmental and pathological roles of TGF-β signaling (Budi et al., 2017).

Adding another turn to the TGF-β signaling pathway, here we describe, for the first time, TβRII-SE, a novel human TβRII splice variant that encodes a truncated soluble isoform of the receptor. Opposite to TβRII and its splice variant TβRII-B, TβRII-SE isoform binds all three TGF-β ligands, in the picomolar affinity range, without participation of additional receptors.

Given the enhanced TGF-β1 signaling in cancer and fibrosis (Derynck and Budi, 2019), TGF-β has become a promising therapeutic target. Here, we checked TβRII-SE functionality in a liver fibrosis animal model. Liver fibrosis is a common stage of all chronic liver diseases (CLD) (Jiang et al., 2018) and is characterized by the excessive synthesis and accumulation of extracellular matrix (ECM) proteins. After liver damage, reparative mechanisms are activated to replace injured hepatocytes. However, if the exposure to the liver injury agent persists over a long time, the continuous wound healing response leads to the destruction of liver architecture and, eventually, results in liver cirrhosis, liver failure, and high risk of developing hepatocarcinoma (HCC) (Malhi and Gores, 2008). Even though for many years it was thought that liver fibrosis was irreversible and progressive, strong evidence has shown that, on the contrary, liver fibrosis is a highly dynamic and reversible process (Povero et al., 2010). TGF-β plays an important role during all phases of the development of liver fibrosis, being responsible for hepatic stellate cell (HSC) activation to myofibroblasts (MFBs) (Fabregat and Caballero-Díaz, 2018), reactive oxygen species (ROS) generation (Liu and Desai, 2015), and ECM production stimulation (Verrecchia and Mauviel, 2007). Therefore, to functionally evaluate the capacity of TβRII-SE to modulate TGF-β effect *in vivo*, we constructed a lentiviral vector encoding TβRII-SE fused in frame with IgG1 Fc domain. Intrahepatic infusion of this vector, in a carbon tetrachloride (CCl_4_)-induced liver fibrosis model, suggested a strong protective effect of TβRII-SE against liver fibrogenesis.

## Results

### TβRII-SE Is a Novel TβRII Splice Variant

**FIGURE 1:**
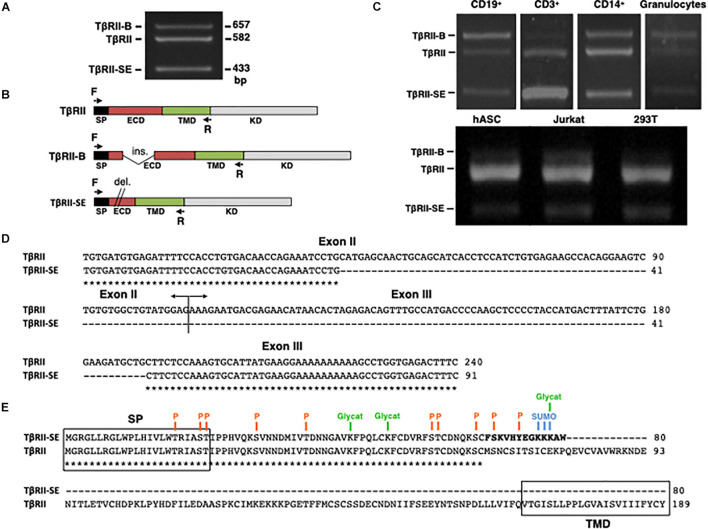
TβRII-SE splice variant is produced by human cells and is able to generate a truncated soluble protein. **(A)** TβRII-SE was originally detected in human lymphocytes as a 433-bp band after end point RT-PCR. **(B)** Primers design to amplify the coding sequence of the endoplasmic reticulum signal peptide (SP), extracellular domain (ECD), and transmembrane domain (TMD) of membrane-bound TβRII splice variants. **(C)** End point RT-PCR of PBMC isolated CD3^+^, CD19^+^, CD14^+^ cells, and granulocytes; and human adipose stromal cells (hASC), immortalized T lymphocyte cell line (Jurkat), and human embryonic kidney cell line containing the SV40 T-antigen (293T). **(D)** Partial nucleotide sequence alignment of TβRII and TβRII-SE depicting the stretch absent in the new splice variant. **(E)** Predicted amino acid sequence alignment of TβRII and TβRII-SE showing the presence of the SP and predicted PTM in both isoforms, and the absence of the transmembrane domain (TMD) in TβRII-SE. C-terminal 13-amino-acid stretch distinctive of TβRII-SE is shown in bold. P, kinase-specific phosphorylation; Glycat, glycation; SUMO, non-consensus sumoylation.

The new TβRII splice variant was first identified as a 433-bp fragment by end point RT-PCR in human peripheral blood-derived T lymphocytes (Figure 1A). The primer pair employed also amplified the cDNA sequence encoding the ER-signal peptide (SP), the extracellular domain (ECD), and the transmembrane domain (TMD) corresponding to the two membrane-bound TβRII splice variants (TβRII and TβRII-B) (Figure 1B). Using this primer pair, we also found the presence of the 433 bp band in CD3^+^, CD19^+^, and CD14^+^ cells isolated by immunomagnetic separation, and in granulocytes obtained from Ficoll density gradient (Figure 1C). In addition, the new band was also detected in primary cultures of adipose-derived mesenchymal stromal cells (hASC), and in the cell lines 293T and Jurkat (Figure 1C).

In comparison to TβRII (Genbank Accession Number NM_003242), DNA sequence analysis revealed, in the new PCR amplified fragment (Genbank Accession Number MW881156), the absence of 149 bp (63 bp of exon II and 86 bp of exon III), indicating alternative splicing (Figure 1D). The predicted amino acid (AA) sequence of this new splice variant showed that the lack of 149 bp alters the coding sequence, creating a small open reading frame (sORF) with a premature stop codon (Figure 1E). Therefore, the new TβRII splice variant has the capacity to encode a protein of 80 AA residues, which includes an ER signal sequence of 23 AA, and a mature protein of 57 AA, lacking the transmembrane domain (TMD) (Figure 1E). Thus, we named the novel TβRII splice variant as TβRII soluble endogenous or TβRII-SE. TβRII and TβRII-SE protein sequence alignment revealed, in the C-terminus of the new isoform, a stretch of 13 AA (FSKVHYEGKKKAW) distinctive of TβRII-SE (Figure 1E). Post-translational modification (PTM) predictions of this novel receptor indicated a putative glycation site (K55), kinase specific phosphorylation (S46 and Y50), and non-consensus sumoylation sites (K53, 54, and 55) (Figure 1E and Supplementary Table 1). The predicted molecular weight of the mature TβRII-SE, without PTM, was 6,532.51 Da with an isoelectric point (pI) of 9.05 (Supplementary Table 1).

### TβRII-SE Splice Variant Encodes a Stable Peptide With Distinctive Binding Properties

**FIGURE 2:**
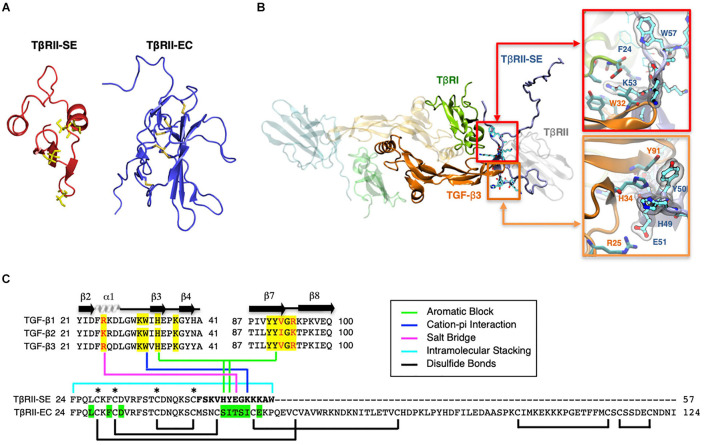
3D modeling reveals unique characteristics of TβRII-SE in comparison to the extracellular domain of membrane-bound TβRII. **(A)** TβRII-SE and TβRII-EC 3D models showing cysteine residues in yellow, and lack of disulfide bonds in TβRII-SE. **(B)** 3D model of mature TβRII-SE superimposed over the crystallographic structure of 2PJY (TβRII/TβRI/TGF-β3) (left panel). The amino acid interactions between TβRII-SE (blue residues) and TGF-β3 (orange residues) are shown magnified in the right upper and lower panels. **(C)** Amino acid sequence alignment of human TGF-β family members (top panel) showing residues interacting with TβRII-EC (yellow shading) and residues interacting with TβRII-SE (color lines linking the bottom panel). Green shading indicates residues in TβRII-EC interacting with TGF-β cognate ligands. Brackets and asterisks indicate cysteine residues forming and not forming disulfide bonds in TβRII-EC and TβRII-SE, respectively.

Based on the TβRII-SE predicted AA sequence, we generated a 3D model using the Robetta server, and the TβRII extracellular (EC) domain as a template (Figure 2A). The stability of the model was confirmed with a long molecular dynamics trajectory. This 3D modeling revealed that TβRII-SE cysteines would not be able to form disulfide bonds as in TβRII-EC (Figure 2A). In addition, to predict how TβRII-SE binds to TGF-β ligands, we superimposed the mature protein model of the new receptor isoform over the crystallographic structure 2PJY, corresponding to the TβRII/TβRI/TGF-β3 complex. In this analysis, we found that TβRII-SE and TGF-β3 establish stable physical contacts. In particular, lysine 53 (K53) of TβRII-SE forms cation–Pi interaction with tryptophan 32 (W32) of TGF-β3, and phenylalanine 60 (F60) of TβRI, whereas tryptophan 57 (W57) of TβRII-SE makes an intramolecular stacking with phenylalanine 24 (F24), promoting a β-fork structure (Figure 2B, upper right panel, and Figure 2C). Similarly, histidine 49 (H49) and tyrosine 50 (Y50) of TβRII-SE form an aromatic stacking with histidine 34 (H34) and tyrosine 91 (Y91) of TGF-β3 (Figure 2B, lower right panel, and Figure 2C). Notably, glutamic acid 51 (E51) of TβRII-SE can form salt bridges with arginine 25 (R25) of TGF-β3, substituting the same interaction between glutamic acid 119 (E119) in the TβRII-EC (extracellular) domain. The interactions present in the distinctive 13 AA stretch of TβRII-SE allow to share with TβRII the same TGF-β binding region (Figure 2C), although both TβRII isoforms are structurally different. These new interactions suggests a more extensive protein–protein interface than TβRII, allowing TβRII-SE to compete for the binding with TβRI and TGF-β ligands, in the trimeric complex.

### TβRII-SE Fc-Tagged Overexpression *in vitro*

**FIGURE 3:**
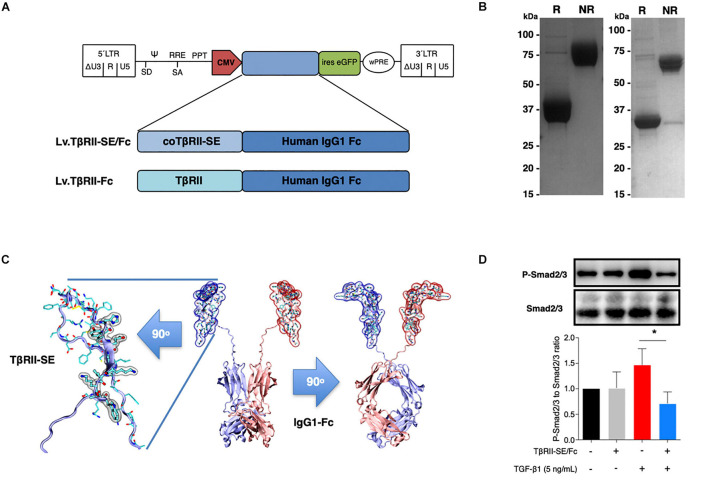
TβRII-SE/Fc overexpressed in human cells is secreted to the extracellular milieu as a disulfide-linked homodimer and inhibits Smad2/3 activation. **(A)** Either coTβRII-SE/Fc or TβRII-EC was ligated to the human IgG1 Fc coding sequence and cloned into a self-inactivating (SIN) bicistronic lentiviral vectors making Lv.TβRII-SE/Fc and Lv.TβRII-Fc, respectively. The proviral lentiviral vectors contain long terminal repeat sequences (LTRs) on both ends, devoid of LTR promoter/enhancer sequences (ΔU3) after reverse transcription. The cytomegalovirus (CMV) promoter was used as internal promoter to drive the expression of either TβRII-SE/Fc or TβRII-Fc. Furthermore, the vectors include the safety-improved woodchuck hepatitis virus post-transcriptional regulatory element (wPRE), a splice donor (SD), a splice acceptor (SA) and the packaging signal (Ψ), together with the Rev responsive element (RRE), and the central polypurine tract (PPT). **(B)** SDS-PAGE and Coomassie blue staining of Protein A/G purified supernatants of TβRII-SE/Fc-overexpressing 293T cells under reducing (R) and non-reducing (NR) conditions. **(C)** Dimeric 3D model of TβRII-SE/Fc using the structures 2PJY (TβRII) and 1L6X (Fc), as templates (middle panel). TβRII-SE is shown magnified and rotated 90° (left panel) with atomic representation wrapped in its solvent-accessible surface area. Amino acids with solvent-accessible surface (highlighted in gray) are part of the protein-to-protein interface. Also, TβRII-SE/Fc 3D model is shown rotated 90° (right panel). **(D)** Representative immunoblotting (top panel) and semi-quantitative densitometry of three independent experiments (bottom panel) of P-Smad2/3 and total Smad2/3 in HCT116 cells overexpressing TβRII-SE/Fc (+) and control (–), in the presence (+) and absence (–) of TGF-β1 stimulation. *p < 0.05.

To generate high levels of TβRII-SE protein for further studies, we aimed to enhance transgene expression in mammalian cells by modifying TβRII-SE coding sequence by inclusion of a Kozak sequence and codon optimization (co) (Supplementary Figure 1). In addition, to ease protein purification and to increase serum half-life of the peptide, we fused coTβRII-SE to human IgG1-Fc domain coding sequences “in frame,” and cloned it into a lentiviral vector to make Lv.TβRII-SE/Fc (Figure 3A).

We next overexpressed TβRII-SE/Fc chimera by transducing 293T cells with the lentiviral vector Lv.TβRII-SE/Fc at high levels (Supplementary Figure 2). Recombinant protein purified by A/G protein chromatography from 293T cells conditioned medium was analyzed by SDS-PAGE gels. Coomassie Blue staining revealed, under reducing and non-reducing conditions, broad bands of approximately 37–38 kDa and 75 kDa, respectively (Figure 3B, left). We also overexpressed TβRII-SE/Fc without N-glycosylation in the CH2 domain of the Fc (Figure 3B, right). Here, we observed bands of 32 and 64 kDa under reducing and non-reducing conditions, respectively. These data were consistent with TβRII-SE/Fc being secreted as a disulfide-linked homodimer, as predicted by a 3D dimeric model (Figure 3C). To determine the potential role of TβRII-SE on downstream TGF-β signaling, we tested its effect on Smad2/3 activation. Lysates from HCT116 cells either transduced with Lv.TβRII-SE/Fc or untransduced (control), in the presence or absence of TGF-β1, were subjected to both anti-Smad2/3 and anti-pSmad2/3 immunoblotting (Figure 3D). TGF-β1 induced Smad2/3 activation in control HCT116 cells, but not in TβRII-SE/Fc-overexpressing cells. These results suggest that TβRII-SE is able to block the TGF-β signaling cascade by trapping TGF-β1, as suggested by 3D modeling, acting as an antagonist.

### TβRII-SE/Fc Binds to the Three TGF-β Isoforms

**FIGURE 4:**
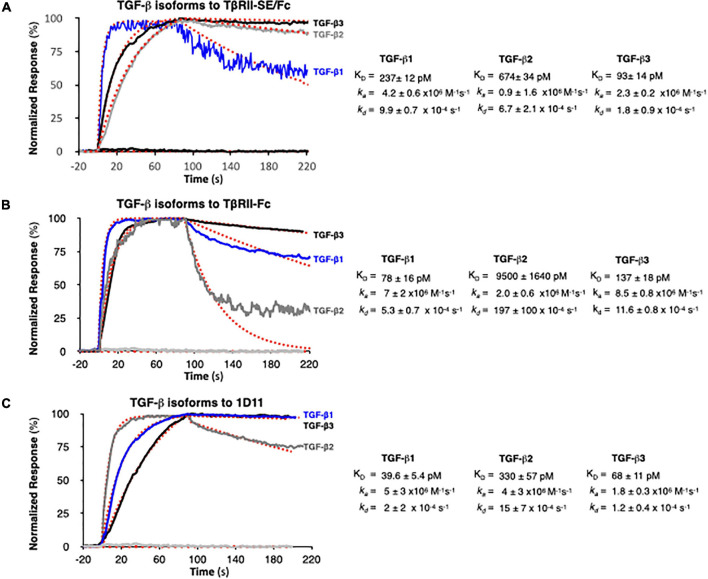
SPR binding profiles of 30 nM TGF-β isoforms to Fc-captured recombinant TβRII-SE/Fc, TβRII-Fc, and 1D11 mAb. Normalized association (*t* = 90 s) and dissociation profiles (*t* = 130 s shown) for **(A)** TβRII-SE/Fc, **(B)** TβRII-Fc, and **(C)** 1D11 mAb complexes with each TGF-β isoform are shown overlayed with the respective global model fits (red dashed lines). Blue: TGF-β1, gray: TGF-β2, and black: TGF-β3. Calculated kinetic parameters are shown to the right for each interaction.

We next examined TGF-β1, -β2, and -β3 binding to TβRII-SE/Fc using surface plasmon resonance (SPR). This is a label-free method that was optimized to rank the kinetics of interaction of the three TGF-β isoforms with TβRII-SE relative to the native ligand, the TβRII receptor, and the pan-isoform specific 1D11 mAb. This is a TGF-β neutralizing antibody with therapeutic efficacy and characterized kinetics of binding. 1D11 binds the three TGF-β isoforms with fast association and slow dissociation kinetics, forming stable homodimers. To assess the kinetics of potentially tight TGF-β-TβRII-SE/Fc complexes, interactions were measured at a flow of 50 μl min^–1^ using 60- to 90-s associations, 15-min dissociations, and low ligand surface densities. We observed rapid and stable kinetics of binding of TGF-β isoforms with TβRII-SE, TβRII, and 1D11 mAb with the exception of TGF-β2 with TβRII, previously described to form fast dissociating complexes. We note that 1:1 interactions that dissociate at rates slower than 1 × 10^–3^ s^–1^ require either longer association phases or higher concentrations to reach a steady state. Consistent with the short associations, and to resolve kinetic differences among TGF-β, we monitored binding at concentrations that were 250-fold the previously reported K_*D*_s of complexes with TβRII-Fc and 1D11, to allow at least one of the TGF-β isoforms binding to reach steady state in 90 s. TβRII-Fc binding TGF-β isoforms reached steady state within 90 s, particularly the lower-affinity TGF-β2, which was not measured to ligand saturation, while TβRII-SE/Fc and 1D11 complexes reached steady state at saturation for at least one isoform, with all interactions displaying slow complex dissociation phases (*k_*d*_s* ranging 2–10 × 10^–4^ s^–1^). Association and dissociation rate constants (*k*_*a*_ and *k*_*d*_) for each TGF-β1, TGF-β2, and TGF-β3 were best estimated using a single step, 1:1 binding model for the two-site binding reactions, which were predicted to be simultaneous with TβRII-SE and TβRII receptor pairs brought into close proximity by the Fc-sequence dimerization. Binding profiles for 30 nM TGF-β1, -β2, and -β3 to TβRII-SE-Fc are overlayed and compared with those obtained with TβRII-Fc and 1D11 in Figures 4A–C, respectively. Fast *k*_*a*_s ranging from 1 to 10 × 10^6^ M^–1^ s^–1^, slow to medium *k*_*d*_s ranging from 2 to 10 × 10^–4^ s^–1^, and maximum binding capacities or R_*max*_ were calculated for all replicate interactions (1:1 model fit, Chi^2^ < 0.02^∗^R_*max*_).

Stoichiometries of complex formation were calculated to explore the mechanistic dominance of homodimeric TGF-β isoforms binding with TβRII-SE/Fc. Surface densities (RU/kDa molecular mass) ranged from 0.6 to 2.4 and resulted in maximum signals (R_*max*_) ranging 45–126 RU for each ligand due to equal accessibility to all binding sites. Maximum binding capacities (or R_*max*_) were used to calculate the stoichiometries of binding after each ligand capture was optimized and ligands were not subjected to any regeneration steps that reduce binding capacity. Supplementary Table 2 shows the theoretical vs. the experimental stoichiometries of binding and was estimated as one TGF-β dimer per TβRII and TβRII-SE bivalent Fc ligand, and two TGF-β dimers per 1D11 as was previously reported in Fab-fragment binding of other neutralizing antibodies that functionally mimic the binding mode of both TβRII and TβRI receptors to the dimer interface. Taken together, these results indicate that TβRII-SE/Fc binds all three TGF-β isoforms with sub-nanomolar affinities that were comparable to the affinities of their complexes with 1D11, but with stoichiometry similar to those of TβRII-Fc binding.

### TβRII-SE/Fc Overexpression Prevents CCl_4_-Induced Liver Fibrosis

**FIGURE 5:**
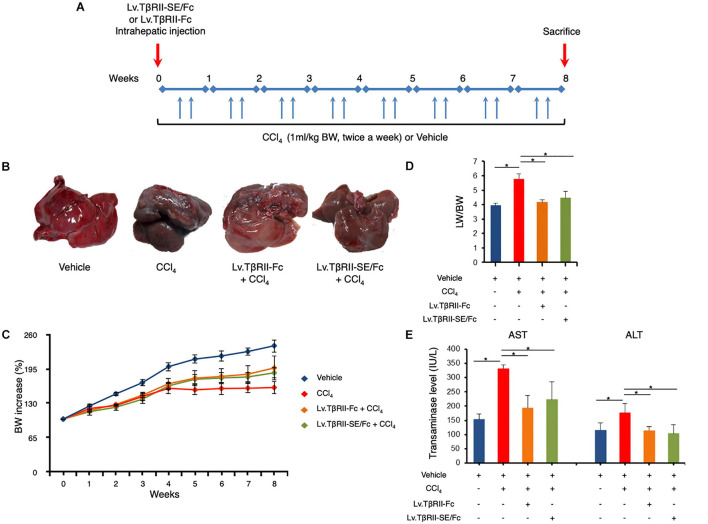
TβRII-SE/Fc overexpression prevents CCl_4_-induced liver damage development. **(A)** Experimental design including lentiviral vector and CCl_4_ administrations. Animals were euthanized by CO_2_ inhalation after 72 h of the last CCl_4_ injection. **(B)** Representative images of liver gross appearance corresponding to rats treated with vehicle, CCl_4_, Lv.TβRII-Fc + CCl_4,_ and Lv.TβRII-SE/Fc + CCl_4_. **(C)** Percentage (%) of both body weight gain (left panel) and **(D)** liver-to-body weight ratio (LW/BW) (right panel) of animals in the different experimental groups. *p < 0.05, Vehicle vs. CCl_4_, or CCl_4_ vs. Lv.TβRII-SE/Fc + CCl_4_. **(E)** Serum activity levels of liver enzymes aspartate aminotransferase (AST) and alanine aminotransferase (ALT) in the different experimental groups. Results are expressed as IU/L. *p < 0.05, Vehicle vs. CCl_4_, or CCl_4_ vs. Lv.TβRII-SE/Fc + CCl_4_. IU: International units.

We next aimed to evaluate the prophylactic effect of TβRII-SE/Fc *in vivo*, in comparison to TβRII-Fc, in a CCl_4_-induced liver fibrosis model as a first approach. To this end, 1 week after intrahepatic administration of either Lv.TβRII-SE/Fc or control Lv.TβRII-Fc, liver fibrosis was induced by chronic administration of the hepatotoxic agent CCl_4_ for 8 weeks (Figure 5A). As controls, we used rats injected with either CCl_4_ only or oil (Vehicle) also for 8 weeks. Liver transduction efficiency was determined by intrahepatic injection of Lv.TβRII-SE/Fc and comparison with mock (PBS) injected livers (Supplementary Figure 3). Macroscopic liver examination showed, in vehicle rats, a reddish color, a smooth surface, and a regular shape (Figure 5B). Conversely, livers of CCl_4_-treated animals showed irregular outlines, opaque color, shrinkage, and unsmooth surfaces. Livers of the Lv.TβRII-SE/Fc + CCl_4_ group were redder, more regular in shape, and with smoother surfaces than the CCl_4_ group, and similar to the Lv.TβRII-Fc + CCl_4_ group. Also, the body weight (BW) of rats was monitored throughout the experiment. Compared to the Vehicle group, CCl_4_ treatment decreased final BW gain (Figure 5C). On the other hand, BW gain was evident after 4 weeks of CCl_4_ administration, in both the Lv.TβRII-SE/Fc + CCl_4_ group and the Lv.TβRII-Fc + CCl_4_ group. In addition, CCl_4_ administration increased liver-to-body weight ratio (LW/BW) compared to the Vehicle group. Contrarily, Lv.TβRII-SE/Fc-injected animals showed LW/BW comparable to that found in both the Vehicle and the Lv.TβRII-Fc + CCl_4_ groups (Figure 5D).

**FIGURE 6:**
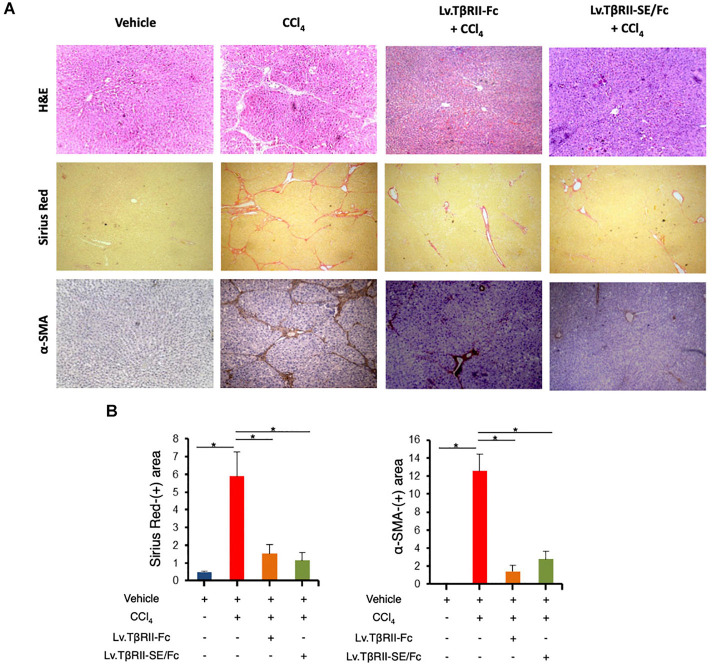
TβRII-SE/Fc overexpression prevents CCl_4_-induced liver fibrosis development and HSC activation. **(A)** Representative images of liver sections stained with either H&E (top panel), Sirius Red (middle panel), and α-SMA immunohistochemistry of animals treated with vehicle, CCl_4_, Lv.TβRII-Fc + CCl_4_, or Lv.TβRII-SE/Fc + CCl_4_. Original magnification × 40. **(B)** Quantification of both liver Sirius Red positive areas (SR^+^) (left panel) and liver α-SMA^+^ expression (right panel) in the same experimental groups. Results are expressed as mean intensity of SR^+^ area. *p < 0.05, Vehicle vs. CCl_4_, or CCl_4_ vs. Lv.TβRII-SE/Fc + CCl_4_. HSC, hepatic stellate cells; H&E, hematoxylin and eosin; α-SMA; alpha-smooth muscle actin.

We also evaluated the effect of TβRII-SE/Fc on CCl_4_-induced liver injury measuring the activity level of serum aspartate aminotransferase (AST) and alanine aminotransferase (ALT). As expected, CCl_4_ administration highly increased liver enzymes above Vehicle group levels. On the other hand, the Lv.TβRII-SE/Fc + CCl_4_ group showed enzyme levels comparable to both the Vehicle and the Lv.TβRII-Fc + CCl_4_ groups (Figure 5E). These results suggest that TβRII-SE/Fc, as well as TβRII-Fc overexpression, prevented CCl_4_-induced liver damage. Moreover, liver section analysis further confirmed this finding. H&E staining revealed that vehicle-injected animals presented livers with a conserved liver architecture, with cords of hepatocytes radiating from the central vein (Figure 6A). Conversely, CCl_4_ administration for 8 weeks led to a disrupted liver architecture, extensive liver injury, and prominent fibrosis. These detrimental effects were clearly attenuated when animals were injected with both Lv.TβRII-SE/Fc and Lv.TβRII-Fc, prior to CCl_4_ treatment (Figures 6A,B). Additionally, we observed, by Sirius Red (SR) staining (Figures 6A,B), extensive deposition of collagen fibers with bridging fibrosis in the CCl_4_ group. Similar to the Lv.TβRII-Fc + CCl_4_ group, the Lv.TβRII-SE/Fc + CCl_4_ group showed reduced fibrosis located around portal areas. In accordance with these observations, we also detected decreased Col1A1 mRNA expression in the livers of the Lv.TβRII-SE/Fc + CCl_4_ group (Supplementary Figure 4).

In addition, we evaluated HSC activation by alpha-smooth muscle actin (α-SMA) immunostaining (Figures 6A,B). In this way, we observed increased α-SMA-positive areas in the CCl_4_ group. Instead, HSC activation was markedly reduced in the Lv.TβRII-SE/Fc + CCl_4_ group, at levels comparable to the Lv.TβRII-Fc + CCl_4_ group. These results suggested that TβRII-SE/Fc, as well as TβRII-Fc overexpression, prevented CCl_4_-induced liver fibrosis. Additionally, we observed by RT-qPCR decreased levels of TNF-α and TGF-β1 mRNAs in the Lv.TβRII-SE/Fc + CCl_4_ group compared to the CCl_4_ + Vehicle group (Supplementary Figure 5). These results suggest an anti-inflammatory effect of TβRII-SE/Fc.

Taken together, our results showed that TβRII-SE is a novel TβRII splice variant encoding a 57-AA mature peptide with distinctive attributes. This new isoform is able to bind all three TGF-β ligands with high affinity, and its Fc-tagged version modulates liver fibrosis *in vivo*.

## Discussion

In the present study, we documented for the first time the presence of a novel splice variant of the Type II TGF-β receptor in human cells. Differently to the known TβRII splice variants that encode membrane-bound receptor isoforms, the newly discovered splice variant, TβRII-SE, encodes a soluble truncated receptor with distinctive structural and binding attributes.

The TβRII–TGF-β ligand interface has been defined by x-ray crystallography. There are 11 residues in TβRII-EC involved in the binding to TGF-β3, which are well conserved in humans, rats, and chicken (Hart et al., 2002). Although three out of 11 residues (Leu27, Phe30, and Asp32) remain in TβRII-SE, they do not seem to participate in the protein–protein interface with TGF-β cognate ligands. However, as revealed by our 3D binding predictions, five residues located in the 13-AA stretch of TβRII-SE generate a novel binding interface, allowing it to compete with TβRII for the binding with TβRI and TGF-β ligands, in the trimeric complex. This prediction was confirmed by SPR analysis, where TβRII-SE showed comparable sub-nanomolar affinity for all three TGF-β ligand complexes.

The crystal structure of the TβRII-EC/TGF-β3 complex, also revealed that 10 residues contacting TβRII are identical in TGF-β1 and β3 (Hart et al., 2002). This is consistent with their high-affinity similarity toward binding TβRII. In the low-affinity ligand TGF-β2, three interfacial positions are conservatively substituted relative to TGF-β1 and β3 (Arg25 > Lys, Val92 > Ile, and Arg94 > Lys). These three residues seem to be responsible for the diminished affinity of TGF-β2 for TβRII, in spite of the conserved nature of the substitutions (Hart et al., 2002). For TβRII-SE, 3D predictions indicated that five residues located in the novel 13-AA stretch were responsible for TGF-β ligand binding. In the TGF-β ligand interface, these residues contact four amino acids, instead of 10 residues in the interface formed by TβRII. One of these amino acids is Arg25 in TGF-β1 and β3 interface, or Arg25 > Lys substituted in TGF-β2. Our SPR results showed that this substitution was not crucial to diminishing TβRII-SE binding affinity for TGF-β2, obtaining values in the picomolar range as with TGF-β1 and TGF-β3.

Conventionally, cells produce cytokine receptors as transmembrane proteins. At the same time, most (if not all) of these receptors are also present in soluble forms (Lokau and Garbers, 2020). Soluble cytokine receptors are the extracellular portions of membrane-anchored receptors. Generally, they retain the capacity to bind their ligands with similar affinity as their membrane-anchored isoforms, fulfilling different functions. Some act agonistically, others are antagonists, and some are even able to accomplish both functions, depending on the biological context and stoichiometry of membrane-bound and unbound receptors (Lokau and Garbers, 2020). Soluble cytokine receptors are mainly generated by two mechanisms: ectodomain shedding and alternative splicing (Lokau and Garbers, 2020). Ectodomain shedding is a process in which the membrane-bound receptor is cleaved directly within the transmembrane domain or in close proximity by a protease, releasing a soluble receptor into the extracellular space. Also, ectodomain shedding controls TGF-β responsiveness by modulating the availability of the cell surface TGF-β receptors. TβRI receptor, but not the TβRII, is cleaved by the transmembrane ADAM17, in response to ERK or p38 MAPK signaling (Derynck and Budi, 2019). Also, betaglycan can be cleaved to release its ectodomain that then functions by sequestering TGF-β. The betaglycan extracellular domain binds all three TGF-β ligands (Derynck and Budi, 2019), with highest affinity for TGF-β2 (Dong et al., 2007). This fact may be relevant for TGF-β2, which binds TβRII with lower affinity than TGF-β1 and TGF-β3 (Derynck and Budi, 2019). On the other hand, soluble receptors can be generated by alternative splicing of mRNA transcripts that usually encode membrane-associated receptors (Lokau and Garbers, 2020). This occurs when the usual exon–intron recognition sequence is not used by the spliceosome machinery, and exons are spliced out instead. As a result, generation of frameshift in the codon sequence produces proteins containing endoplasmic reticulum-signal peptides and lacking transmembrane domains that are consequently secreted (Lokau and Garbers, 2020). This is the case for the TβRII-SE splice variant, where 149 nucleotides corresponding to parts of exon II and exon III are spliced out in the mature mRNA. The lack of this sequence generates a frameshift in the codon sequence resulting in a truncated soluble receptor with an endoplasmic reticulum signal peptide and a distinctive stretch of 13 amino acid residues in the C-terminus. Additionally, we showed by SPR that TβRII-SE binds all three TGF-βs, with high affinity (picomolar range). While betaglycan also binds all three TGF-βs, SPR analysis indicated that its soluble form has lower affinity for the three TGF-β isoforms (nanomolar range) (Mendoza et al., 2009) than TβRII-SE. Although both receptors seem to have overlapping binding targets, the mechanistic role of TβRII-SE remains to be elucidated.

There are several Fc-fusion drugs already in the pharmaceutical market (Czajkowsky et al., 2012). Fc-based fusion proteins are composed of a peptide fused to an immunoglobin Fc domain. Due to its interaction with the neonatal Fc-receptor (FcRn) (Roopenian and Akilesh, 2007), and slower renal clearance (Kontermann, 2011), the presence of the Fc domain increases the plasma half-life of the fusion protein and prolongs its therapeutic activity. Biophysically, the Fc domain independent folding can improve the stability and solubility *in vivo* and *in vitro* of the partner peptide. Additionally, the Fc region facilitates its purification by using protein-G/A affinity chromatography during manufacture, in a cost-effective manner (Carter, 2011). Protein-G/A purified TβRII-SE/Fc allowed us to initially determine the apparent molecular mass of the monomer as a broad band of approximately 37 kDa. This observation is in agreement with the sum of the predicted molecular weight of TβRII-SE (6.5 kDa), plus the apparent molecular mass of the IgG1-Fc domain (31–32 kDa) in SDS-PAGE under reducing conditions, as a result of glycosylation (Tandale et al., 2020).

Tissue fibrosis affects many organs including the liver, skin, heart, kidney, and lung, and is a leading cause of morbidity and mortality worldwide (Friedman et al., 2013). TGF-β is considered a master regulator of ECM accumulation and, consequently, a key driver of fibrosis (Walton et al., 2017). In the liver, TGF-β is responsible for transdifferentiation of quiescent HSC to a MFB phenotype. MFBs are characterized by the expression of α-SMA, and they are the main producers of fibrogenesis mediators and ECM proteins (Fabregat and Caballero-Díaz, 2018). Given the prominent role of TGF-β in hepatic fibrogenesis, we checked TβRII-SE functionality in CCl_4_-induced liver fibrosis rats.

In this model, we observed that hepatic overexpression of TβRII-SE/Fc diminished liver damage and attenuated liver fibrosis development to levels shown by TβRII-Fc. Although these results demonstrated the prophylactic effect of TβRII-SE/Fc in liver fibrosis, they set the ground to evaluate the therapeutic effect of the approach. In this respect, preliminary results showed that administration of Lv.TβRII-SE/Fc after liver fibrosis induction with CCl_4_ alleviates liver injury and fibrosis deposition in rats (data not shown). Moreover, we have evaluated the therapeutic effect of purified TβRII-SE/Fc by intravenous (IV) biweekly injection in rats after 2 weeks of CCl4 treatment, observing statistically significant differences in fibrosis score, inflammatory cell infiltrate, and oval cell hyperplasia compared to CCL4-treated rats injected IV with vehicle (data not shown).

It is well known that the profibrogenic TGF-β1 can exert its functions through canonical pathways that ultimately result in the expression of collagen type 1 and α-SMA, among others. Thus, our results suggest that CCl_4_ administration activated the canonical pathway since TGF-β1 and its downstream effectors α-SMA and Col1A1 were increased in these animals, whereas TβRII-SE/Fc delivery decreased both of them in rat livers. However, we cannot discard the idea that non-canonical pathways might also be modulated by TβRII-SE/Fc to exert its protective effect. Our results suggest that TβRII-SE/Fc expression diminishes canonical profibrogenic signals that conduce to HSC activation. Chronic inflammation has an important role in liver fibrosis pathogenesis. Indeed, there exists a crosstalk between inflammatory cells and HSC, in which the production of inflammatory mediators leads to HSC activation that, then, results in excessive ECM deposition. In accordance, our results suggest that TβRII-SE/Fc expression decreases liver inflammation that, in turn, can limit HSC activation and subsequent liver injury.

In order to elucidate the physiological relevance of the native peptide TβRII-SE, further studies are needed and some of them are underway. In the meantime, we wanted to assess whether the peptide encoded by the new splice variant was capable to act similarly to TβRII regarding its anti-fibrotic effect. Thus, for functional tests, we used a lentiviral vector encoding TβRII-SE/Fc instead of TβRII-SE without Fc because the former allowed us a direct comparison with the therapeutically relevant chimeric soluble TβRII (TβRII-Fc). This recombinant protein, also named soluble TβRII (sTβRII), or Fc:TβRII, has been constructed by fusion of genes encoding the N-terminal (extracellular) fragment of TβRII and the Fc domain of human IgG. This chimeric protein has demonstrated efficacy for selective blocking of TGF-β family ligands in pathological conditions (Zhou et al., 2010; Yung et al., 2016), including liver fibrosis (Dooley and ten Dijke, 2012). However, its therapeutic potential is isoform-selective, as TGF-β1 and TGF-β3 are known to bind TβRII receptor dimers with high affinity, but not TGF-β2 (Hinck et al., 2016), as we confirmed here by SPR analysis. TGF-β family proteins are secreted and function as disulfide-linked homodimers or heterodimers (Derynck and Budi, 2019). Therefore, the stoichiometry of TGF-β dimer to TβRII dimer is known to be 1:1. Our SPR analyses confirmed this observation for TβRII-Fc and indicated the same stoichiometry for TβRII-SE/Fc.

Here, we also show that TβRII-SE/Fc is as effective at binding all three TGF-β isoforms as 1D11 mAb. This is a mouse pan-TGF-β neutralizing antibody (Dasch et al., 1989), that is, the parent antibody of the humanized and optimized version GC-1008 (Ledbetter et al., 2010). The crystal structure of the GC-1008/TGF-β3 complex showed that some residues of the FWH3 and all CDR loops of the heavy chain are involved in the binding. The Fab recognizes parts of both TGF-β3 molecules in the homodimer, not only residues from a single monomer (Grütter et al., 2008). The total binding area of TGF-β to the antibody consists of two identical binding interfaces on the surface of the TGF-β3 homodimer. Our binding data confirmed that 1D11 binds a dimer per Fab as reflected in the calculated stoichiometry of TGF-β homodimer per antibody of 2:1.

In this article, we demonstrate that TβRII-SE/Fc and TβRII-Fc share similarities, such as binding affinity to TGF-β isoforms, *in vitro* blockade of TGF-β1 signaling, and *in vivo* prevention/inhibition of liver fibrosis. Thus, we hypothesized that, like TβRII-Fc, TβRII-SE/Fc would be able to act as a ligand trap, despite the fact that further studies must be conducted to clarify its mechanism of action.

TGF-β2, although less characterized than TGF-β1, also displays potent profibrotic activity. TGF-β2 is increased in late stages of liver fibrosis associated to hepatitis C virus (HCV) infection in patients (Sancho et al., 2012), is accumulated in the bile ducts in human fibrotic liver disease (Milani et al., 1991), and is elevated in the aqueous humor of patients with glaucoma (Wordinger et al., 2014). Information regarding TGF-β3 is scarce in the literature. *In vitro*, TGF-β3 has been shown to exert profibrotic effects on fibroblasts (Serini and Gabbiana, 1996; Coker et al., 1997). Thus, the fact that TβRII-SE binds all three TGF-β ligands with high affinity could offer an alternative strategy to treat fibrosis-related diseases. Also, increased expression of TGF-β1, -β2, and -β3 has been reported in glioblastoma, breast cancer, and colorectal cancer. In particular, high TGF-β2 expression is associated with poor prognosis of advanced lung cancer, gliomas, and skin squamous carcinoma (Takahashi et al., 2020). Therefore, TβRII-SE/Fc may be a promising tool for the prevention and treatment of pathological conditions caused by TGF-β upregulation, especially TGF-β2.

## Materials and Methods

### Cell Culture

Human adipose-derived mesenchymal stromal cells (hASC), purified as described (Rodríguez et al., 2015), and human cell lines A549, Jurkat, 293T, and HCT116 were cultured in DMEM supplemented with 10% FBS and 1% penicillin/streptomycin, in a humidified 5% CO_2_ incubator at 37°C.

### Purification of Leukocyte Subsets

Granulocytes, lymphocytes, and monocytes were isolated from heparinized human peripheral blood by Ficoll-Paque^TM^ PLUS (GE Healthcare Bio- Sciences AB, Uppsala, Sweden) gradient centrifugation. After centrifugation, two fractions were obtained: one containing granulocytes/erythrocytes and another with peripheral blood mononuclear cells (PBMCs). To obtain granulocytes, erythrocytes were lysed with KCl 0.6 M. PBMCs were labeled with anti-CD3^+^, -CD14^+^, and -CD19^+^ monoclonal antibodies conjugated with magnetic microbeads (Miltenyi Biotech, Bergisch Gladbach, Germany) and separated using MS columns in a MiniMACS magnet (Miltenyi Biotech, Bergisch Gladbach, Germany). Viable cells were determined by Trypan blue dye exclusion and counted in a hemocytometer. The purity of CD19^+^, CD3^+^, and CD14^+^ cells was determined by flow cytometric analysis using a FACSCalibur flow cytometer (BD Biosciences, San Jose, CA, United States). Cell sub-populations were homogenized in RNA Lysis Buffer (SV Total RNA Isolation System, Promega Corporation, Madison, WI, United States) and stored at −80°C until RNA purification.

### End Point RT-PCR

Total RNA from different primary cultures and cell lines was isolated using the SV Total RNA Isolation System, and cDNA was generated using 1 mg of RNA, M-MLV Reverse Transcriptase, and oligo dT_(15)_ primers, according to the indications stated by the manufacturer (Promega Corporation, Madison, WI, United States). To simultaneously detect the different splice variants of the TβRII receptor, PCR amplification was performed in the presence of Expand High Fidelity polymerase (Roche Diagnostics GmbH, Mannheim, Germany), 0.2 mM dNTPS, and 0.5 μM of each primer (forward: 5′ACCGGTATGGGTCGGGGGCTGCTC3′ and reverse: 5′GT CGACTCAGTAG CAGTAGAAGATG3′) for 35 cycles using the following PCR conditions: 1 min at 95°C, 1 min at 55°C, and 1 min at 95°C.

### RT-qPCR

Total RNA from liver tissues was isolated using the UltraClean^®^ Tissue and Cells RNA Isolation Kit (Mobio Laboratories, Inc., Carlsbad, CA, United States), cDNA was generated using 1 mg of RNA, M-MLVReverse Transcriptase, and oligo dT_(15)_ primers, according to the indications stated by the manufacturer (Promega Corporation, Madison, WI, United States). PCR amplification was performed in the presence of PowerUp^TM^SYBR^TM^ Green Master Mix (Thermo Fisher Scientific, Waltham, MA, United States) according to the manufacturer’s instructions using the following oligonucleotides: COL1A1 forward primer CAGTCGATTCACCTACAGCACG, reverse primer GGGATGGAGGGAGTTTACACG; TGF-β1 forward primer GGAGAGCCCTGGATACCAACT, reverse primer AGGACCTTGCTGTACTGTGTGT; TNF-α forward primer GCCCAGACCCTCACACTCAG, reverse primer CGCTTGGTGGTTTGCTACGAC; β-actin forward primer AG GGTGTGATGGTGGGTATGG, reverse primer GTGT GGTGCCAAATCTTCTCCA. The specificity of PCR products was confirmed by melting curve analysis. The levels of the transcripts were normalized to β-actin, which was used as a reference gene. Relative quantification of gene expression was determined by the comparative CT method.

### DNA Sequencing

PCR fragments were cloned into the pGEM-T Easy plasmid (Promega Corporation, Madison, WI, United States) under the conditions established by the manufacturers and *E. coli* transformation. TβRII PCR fragments were sequenced by using M13 forward and reverse primers using a capillary automatic sequencer model ABI3130XL (Applied Biosystems, United States) at the Genomic Unit of the Biotechnology Institute, INTA, Consorcio Argentino de Tecnología Genómica (CATG) (PPL Genómica, MINCyT).

### TβRII-SE Codon Optimization and Human IgG1 Fc Chimeric Fusion Protein

TβRII-SE was codon optimized (co) together with the deletion of the stop codon. This product also included a Kozak sequence, and an *Age*I site and *EcoR*V at the 5′ and 3′ end, respectively (Epoch Biolabs Inc., Missouri City, TX, United States). The human IgG1 Fc coding sequence was obtained by RT-PCR from peripheral blood leucocyte mRNA using specific oligonucleotides as primers (forward: 5′-AGATCTGACAAAACTCACACATGC-3′ and reverse: 5′-GATATCTTTACCCGGAGACAGG-3′), containing a *Bgl*II recognition site (forward primer) and an *Eco*RV site (reverse primer). The coTβRII-SE sequence (258 bp) was fused *in frame* with the coding sequence of the IgG1 Fc domain (693 bp) to generate the coTβRII-SE/Fc fusion cDNA of 951 bp.

### Lentiviral Vector Production

The cDNA encoding TβRII-SE with IgG1 Fc was cloned into the pRRLsin18.cPPT.WPRE lentiviral vector, generating the vector pRRLsin18.cPPT.CMV-coTβRII-SE/Fc.ires.eGFP.WPRE (Lv.TβRII-SE/Fc). As control, we cloned the coding sequence of TβRII extracellular domain (TβRII-EC) fused in frame with IgG1 Fc to generate the vector pRRLsin18.cPPT.CMV-TβRII-Fc.ires.eGFP.WPRE (Lv.TβRII-Fc). Vesicular Stomatitis Virus G protein-pseudotyped lentiviruses (VSV-G) were generated by transient transfection of the transfer vectors together with the envelope plasmid (pCMV-VSVG), the packaging plasmid (pMDLg/pRRE), and the Rev plasmid (pRSV-REV), into the 293T cell line, as previously described (Noyan et al., 2012). Cell supernatants were harvested once every 12 h, for 48 h, and frozen in aliquots. Viral titers were determined by transducing A549 cells, yielding 10^7^ TU (transducing units) per milliliter.

### Smad2/3 Activation and Immunoblotting

Human colorectal cancer-derived cell line HCT116 cells were transduced with Lv.TβRII-SE/Fc at MOI 200, in the presence of 8 μg/ml polybrene (Millipore Sigma, Burlington, MA, United States). HCT116 cells (1 × 10^6^) overexpressing TβRII-SE/Fc or control were seeded in 60-mm cell culture dishes and starved for 24 h in DMEM. After that, cells were incubated in DMEM ± 5 ng/ml TGF-β1 for 1 h. Cells were lysed in RIPA buffer (Millipore Sigma, Burlington, MA, United States) supplemented with 1 mM PMSF and quantified by Bradford Assay. Proteins were separated by electrophoresis on 10% SDS-PAGE gels and electrotransferred onto Immobilon-polyvinylidene difluoride membranes (Millipore Sigma, Burlington, MA, United States). After blocking with 5% non-fat milk, membranes were probed with antibodies for P-Smad2/3 (sc-11769) and Smad2/3 (sc133098) (Santa Cruz Biotechnology, Inc., Dallas, TX, United States) at 4°C overnight and then incubated with horseradish peroxidase (HRP)-conjugated secondary anti-mouse or rabbit antibodies (Thermo Fisher Scientific, Waltham, MA, United States). Protein expression was detected by using an enhanced chemiluminescence (ECL) system (Thermo Fisher Scientific, Waltham, MA, United States). Densitometry was performed using ImageJ software (National Institutes of Health, Bethesda, MD, United States).

### TβRII-SE/Fc-Fusion Recombinant Protein Production and Purification

Human 293T cells were transduced either with Lv.TβRII-SE/Fc or Lv.TβRII-Fc at a MOI of 70, in the presence of 8 μg/ml polybrene (Millipore Sigma, Burlington, MA, United States). Forty-eight hours after transduction, cells were harvested, washed in phosphate-buffered saline (PBS) supplemented with 10% FBS, and the percentage of eGFP-positive cells was determined by flow cytometry on a FACSCalibur device (BD Biosciences, San Jose, CA, United States).

For the production of TβRII-SE/Fc and TβRII-Fc, lentivirally transduced 293T cells were cultured for 48 h in serum-free DMEM supplemented with Protease Inhibitor Cocktail (1/800) (Millipore Sigma, Burlington, MA, United States). Subsequently, the conditioned media was clarified by centrifugation at 3,500 rpm and filtrated through 0.22-μm filters. The recombinant protein was concentrated by centrifugation in Amicon^®^ Ultra-15-30K (Millipore Sigma, Burlington, MA, United States), purified on protein A/G columns (NAb^TM^ Spin Kit, Thermo Scientific, Rockford, IL, United States) following the instructions of the manufacturer, and stored at −80°C.

### TβRII-SE 3D Modeling and Molecular Dynamic Simulation

We generated a preliminary 3D model of the TβRII-SE peptide structure using the Robetta server (Kim et al., 2004). Robetta produces protein models using comparative modeling and *ab initio* methods. Domains without a detectable template are modeled using the Rosetta *de novo* algorithm. In the initial stage, Robetta identified the extracellular (EC) domain of human TβRII [Protein Data Bank of the Research Collaboratory for Structural Bioinformatics (RCSB-PDB): 1PLO_A] as a confident template for TβRII-SE modeling using the method “Ginzu,” and used it as a template to produce models with a comparative modeling protocol. Robetta carried out multiple independent simulations to generate thousands of models, and finally, five of them were selected from this ensemble by applying different variants of the Rosetta energy function. We selected the best model with the lowest energy informed by Robetta and proved its stability along a 1,000-ns-long molecular dynamics trajectory during which the model maintained its fold and Secondary Structure Elements. Thus, we used this model for further analysis (see Supplementary Materials and Methods for further details).

To predict the binding of TβRII-SE to its TGF-β ligands, we used the ternary complexes of the structures PDB code: 3KFD (TβRII/TβRI/TGF-β1) and PDB code: 2PJY (TβRII/TβRI/TGF-β3) deposited in the Protein Data Bank (PDB), as templates. In addition, TβRII-SE/Fc fusion protein was modeled using the structures PDB code: 2PJY (TβRII) and PDB code: 1L6X (Fc), as templates.

### SPR Assays

Surface plasmon resonance experiments were performed using a Biacore 3000 system (Cytiva Life Sciences, Marlborough, MA, United States). All assays were performed using CM5 sensor chips (Cytiva Life Sciences, Marlborough, MA, United States) derivatized as described previously (Canziani et al., 2004), with anti-hu Fc mixed with equimolar anti-mu Fc antibodies (cat # 109-005-008 and 115-005-008, respectively; Jackson ImmunoResearch, West Grove, PA, United States) at pH 5.0, obtaining four ∼6,000 RU anti-Fc surfaces including reference surface or non-specific binding control. Human Fc recombinant proteins TβRII-SE/Fc (64 kDa), TβRII-Fc (124 kDa), and murine mAb 1D11 (150 kDa) (Bedinger et al., 2016) (Thermo Fisher Scientific, Waltham, MA, United States) were each captured on individual flow cells to 100–140 RU. Captures were designed with a wait time of 60 s after the association to reach a stable signal, and the capture antibodies reference flow cell had no ligand captured. TGF-β1, TGF-β2, and TGF-β3 (PeproTech, Cranbury, NJ, United States), were individually injected over the four flow cells over a range of concentrations prepared by serial twofold or threefold dilutions, spanning from 0.4 to 30 nM, or at two, fivefold apart, concentrations in higher-throughput tests depending on the experimental design, all at a flow rate of 50 μl min^–1^. Non-specific binding of the TGF-β isoforms to the reference capture antibody surfaces was initially detected and minimized by increasing 20-fold the surfactant concentration in the sample and running buffers from 0.005 to 0.1% as previously recommended (Bedinger et al., 2016). Multiple-cycle kinetics were programmed using an association time of 90 s and a dissociation time of 300 s. Capture surfaces were regenerated using three 3-s, 100 mM phosphoric acid (pH 2.0). The assessment of dissociation for 900 s allowed us to confirm 10% loss of complex to measure the slower dissociation rates to calculate the dissociation constants (*k*_*d*_). Running buffer samples were also injected using the same method program for background noise and capture level drift subtraction (double reference). All data were fitted to a 1:1 binding model using Biacore Evaluation Software 3.1 (Cytiva Life Sciences, Marlborough, MA, United States) and Scrubber 2.0c (BioLogic Software, Australia) after double referencing, where *n* = 6 independent experiments using three capture chips, presented kinetic constants ± SEM, and thermodynamic constant K_*D*_ ± SEM, 95% CI (confidence interval). Mass ratios of approximately 2.5:1, 5:1, or 6:1 for TβRII-SE/Fc, TβRII-Fc, and 1D11, respectively, were used to estimate the stoichiometries of complex formation.

### Animals

Male Wistar rats (150–200 g) were housed at the Mar del Plata National University Laboratory Animal Unit at a mean constant temperature of 22°C with a 12-h light–dark cycle, and free access to standard pellet chow and water. All experiments were performed according to the “Guide for the Care and Use of Laboratory Animals” and approved by the Institutional Animal Care and Use Committee (CICUAL) of Mar del Plata National University.

### *In vivo* Liver Transduction and Liver Fibrosis Induction

Liver fibrosis was induced by intraperitoneal injection (i.p.) of 1 ml/kg BW of CCl_4_ in oil (1:1) twice a week for 8 weeks. A week before liver fibrosis induction, rats received an intrahepatic injection (200 μl) of either Lv.TβRII-SE/Fc or Lv.TβRII-Fc (5–10 × 10^7^ TU/ml) (*N* = 6 in each group). Lentiviral vectors were directly injected, after a small incision in the abdomen, into the exposed livers of anesthetized rats. Control animals received an i.p. injection of either CCl_4_ or oil (vehicle) (*N* = 6 each group). Animals were weekly weighed, and measurements were used to calculate the percentage of BW gain. After 8 weeks, animals were euthanized, and livers were weighed to calculate the LW/BW. Livers were fixed in 10% neutral buffered formalin for histological analysis. Serum was also collected for further biochemical analysis.

### Biochemical Parameters

Serum AST and ALT activity levels were measured with an automatic analyzer BT300 plus (Biotecnica Instruments S.p.A., Rome, Italy) according to the manufacturer.

### Histological Analysis

Livers fixed in 10% neutral buffered formalin were embedded in paraffin. Liver sections (5 μm) were stained with Hematoxylin and Eosin staining (H&E) for liver architecture visualization. To assess fibrosis, liver sections were stained with Sirius Red staining (0.1%). Quantification of Sirius Red-positive (SR^+^) areas was performed in at least 10 fields per histological section using the ImageJ software. Results were expressed as mean intensity of SR^+^ area per field.

### Immunohistochemical Analysis

For immunohistochemical analysis, 5-μm sections were deparaffinized and rehydrated. Endogenous peroxidase was blocked by the addition of 3% H_2_O_2_, in methanol. Antigen retrieval was performed using the heat induced epitope retrieval (HIER) method with a citrate buffer of 0.1 M, pH 6. Tissue sections were then incubated with rabbit anti-α-smooth muscle actin (anti-α-SMA, 1:500, Cell Signaling Technology, Danvers, MA, United States) overnight at 4°C. After two washes with PBS, slides were incubated with HiDef Detection amplifier Mouse and Rabbit reagent (Cell Marque, Rocklin, CA, United States) for 10 min, at room temperature. Sections were further washed with PBS and incubated with HiDef Detection HRP Polymer Detector solution (Cell Marque, Rocklin, CA, United States) for 10 min at room temperature. Finally, sections were washed twice with PBS, incubated with the DAB Chromogen kit (Cell Marque, Rocklin, CA, United States) for 5 min at room temperature, and counterstained with Hematoxylin. Dehydrated sections were mounted, and microphotographed on a light microscope, Nikon Eclipse E200. Quantification of α-SMA-positive (α-SMA^+^) areas was performed through use of Fiji software. Results were expressed as the mean intensity of α-SMA^+^ area per field.

### Statistical Analysis

Statistical analysis was performed using GraphPad Prism Version 7.0 (GraphPad Software, San Diego, CA, United States). Data are shown as mean ± SD. Statistical differences among groups were performed using two-way ANOVA and the multiple comparison *post hoc* test by Fisher. For all analyses, a *p*-value < 0.05 was considered statistically significant.

## Data Availability Statement

The datasets presented in this study can be found in online repositories. The names of the repository/repositories and accession number(s) can be found below: GenBank, MW881156.

## Ethics Statement

The animal study was reviewed and approved by Institutional Animal Care and Use Committee (CICUAL) of Mar del Plata National University.

## Author Contributions

ANC and RAD conceived and designed the study. MSB, ALC, AC, TMR, GPB, AMM, SME, SC, AR, and GC performed the analyses. RAD wrote the manuscript. ALC, GPB, AMM, and GC wrote sections of the manuscript. All authors contributed to manuscript revision and read and approved the submitted version.

## Conflict of Interest

AC, TMR, ANC, and RAD are co-inventors of the patent family “Isoform of the TGF-beta receptor II,” US10233227B2 (granted in United States), EP3082846B1 (granted by the European Patent Office), ES2749615T3 (granted in Spain), and AR098827A1 (pending in Argentina). TMR, ANC, ALC, MSB, AR, and RAD are co-inventors of the patent application “TGF-β receptor II isoform, fusion peptide, methods of treatment and methods *in vitro*,” US11072647B2 (granted in United States). Patents are owned by CONICET and Fundación Articular, and were licensed to RAD BIO S.A.S. by Intellectual property license agreement 2019-890-APN-DIR#CONICET. AR has shareholder equity of RAD BIO S.A.S. RAD is the co-founder and shareholder of RAD BIO S.A.S. The remaining authors declare that the research was conducted in the absence of any commercial or financial relationships that could be construed as a potential conflict of interest.

## Publisher’s Note

All claims expressed in this article are solely those of the authors and do not necessarily represent those of their affiliated organizations, or those of the publisher, the editors and the reviewers. Any product that may be evaluated in this article, or claim that may be made by its manufacturer, is not guaranteed or endorsed by the publisher.

## References

[B1] Bedinger D. Lao L. Khan S. Lee S. Takeuchi T. Mirza A. M. (2016). Development and characterization of human monoclonal antibodies that neutralize multiple TGFβ isoforms. *mAbs* 8 389–404. 10.1080/19420862.2015.1115166 26563652 PMC4966579

[B2] Budi E. H. Duan D. Derynck R. (2017). Transforming growth factor-β Recep-tors and Smads: regulatory complexity and functional versatility. *Trends Cell Biol.* 27 658–672. 10.1016/j.tcb.2017.04.005 28552280

[B3] Canziani G. A. Klakamp S. Myszka D. G. (2004). Kinetic screening of antibod-ies from crude hybridoma samples using Biacore. *Anal Biochem.* 325 301–307. 10.1016/j.ab.2003.11.004 14751265

[B4] Carter P. J. (2011). Introduction to current and future protein therapeutics: a protein engineering perspective. *Exp. Cell Res.* 317 1261–1269. 10.1016/j.yexcr.2011.02.013 21371474

[B5] Chaikuad A. Bullock A. N. (2016). Structural basis of intracellular TGF-β Signal-ing: receptors and smads. *Cold Spring Harb. Perspect. Biol.* 8:a022111. 10.1101/cshperspect.a022111 27549117 PMC5088531

[B6] Coker R. K. Laurent G. J. Shahzeidi S. Lympany P. A. du Bois R. M. Jeffery P. K. (1997). Transforming growth factors-beta 1, -beta 2, and -beta 3 stimulate fibroblast procollagen production in vitro but are differentially ex-pressed during bleomycin-induced lung fibrosis. *Am. J. Pathol.* 150 981–991. 9060836 PMC1857875

[B7] Czajkowsky D. M. Hu J. Shao Z. Pleass R. J. (2012). Fc-fusion proteins: new developments and future perspectives. *EMBO Mol. Med.* 4 1015–1028. 10.1002/emmm.201201379 22837174 PMC3491832

[B8] Dasch J. R. Pace D. R. Waegell W. Inenaga D. Ellingsworth L. (1989). Monoclonal antibodies recognizing transforming growth factor-beta. Bioactivity neutralization and transforming growth factor beta 2 affinity purification. *J. Immunol.* 142 1536–1541. 2537357

[B9] del Re E. Babitt J. L. Pirani A. Schneyer A. L. Lin H. Y. (2004). In the ab-sence of type III receptor, the transforming growth factor (TGF)-beta type II-B receptor requires the type I receptor to bind TGF-beta2. *J. Biol. Chem* 279 22765–22772. 10.1074/jbc.m401350200 14996829

[B10] Derynck R. Budi E. H. (2019). Specificity, versatility, and control of TGF-β family signaling. *Sci. Signal.* 12:eaav5183. 10.1126/scisignal.aav5183 30808818 PMC6800142

[B11] Dong M. How T. Kirkbride K. C. Gordon K. J. Lee J. D. Hempel N. (2007). The type III TGF-beta receptor suppresses breast cancer progression. *J. Clin. Invest.* 117 206–217. 17160136 10.1172/JCI29293 PMC1679965

[B12] Dooley S. ten Dijke P. (2012). TGF-β in progression of liver disease. *Cell Tissue Res.* 347 245–256. 10.1007/s00441-011-1246-y 22006249 PMC3250614

[B13] Ehrlich M. Gutman O. Knaus P. Henis Y. I. (2012). Oligomeric interactions of TGF-β and BMP receptors. *FEBS Lett.* 586 1885–1896. 10.1016/j.febslet.2012.01.040 22293501

[B14] Fabregat I. Caballero-Díaz D. (2018). Transforming growth factor-β-induced cell plasticity in liver fibrosis and *Hepatocarcinogenesis*. *Front. Oncol.* 8:357. 10.3389/fonc.2018.00357 30250825 PMC6139328

[B15] Friedman S. L. Sheppard D. Duffield J. S. Violette S. (2013). Therapy for fi-brotic diseases: nearing the starting line. *Sci. Transl. Med.* 5:167sr1. 10.1126/scitranslmed.3004700 23303606

[B16] Grütter C. Wilkinson T. Turner R. Podichetty S. Finch D. McCourt M. (2008). A cytokine-neutralizing antibody as a structural mimetic of 2 receptor interactions. *Proc. Natl. Acad. Sci. U.S.A.* 105 20251–20256. 10.1073/pnas.0807200106 19073914 PMC2600578

[B17] Hart P. J. Deep S. Taylor A. B. Shu Z. Hinck C. S. Hinck A. P. (2002). Crystal structure of the human TbetaR2 ectodomain–TGF-beta3 complex. *Nat. Struct. Biol.* 9 203–208. 11850637 10.1038/nsb766

[B18] Hata A. Chen Y. G. (2016). TGF-β Signaling from receptors to smads. *Cold Spring Harb. Perspect. Biol.* 8:a022061. 10.1101/cshperspect.a022061 27449815 PMC5008074

[B19] Heldin C. H. Moustakas A. (2016). Signaling receptors for TGF-β family mem-bers. *Cold Spring Harb. Perspect. Biol.* 8:a022053. 10.1101/cshperspect.a022053 27481709 PMC4968163

[B20] Hinck A. P. Mueller T. D. Springer T. A. (2016). Structural biology and Evolu-tion of the TGF-β family. *Cold Spring Harb. Perspect. Biol.* 8:a022103. 10.1101/cshperspect.a022103 27638177 PMC5131774

[B21] Hirai R. Fijita T. (1996). A human transforming growth factor-beta type II receptor that contains an insertion in the extracellular domain. *Exp. Cell Res.* 223 135–141. 8635485 10.1006/excr.1996.0066

[B22] Huse M. Chen Y. G. Massagué J. Kuriyan J. (1999). Crystal structure of the cytoplasmic domain of the type I TGF beta receptor in complex with FKBP12. *Cell* 96 425–436. 10.1016/s0092-8674(00)80555-3 10025408

[B23] Jiang H. Zheng T. Duan T. Chen J. Song B. (2018). Non-invasive in vi-vo imaging grading of liver fibrosis. *J. Clin. Transl. Hepatol.* 6 198–207. 29951365 10.14218/JCTH.2017.00038 PMC6018309

[B24] Kim D. E. Chivian D. Baker D. (2004). Protein structure prediction and analysis using the Robetta server. *Nucleic Acids Res.* 32 W526–W531. 15215442 10.1093/nar/gkh468 PMC441606

[B25] Kontermann R. E. (2011). Strategies for extended serum half-life of protein thera-peutics. *Curr. Opin. Biotechnol.* 22 868–876. 10.1016/j.copbio.2011.06.012 21862310

[B26] Ledbetter S. R. Hart C. P. Holgate R. G. Jermutus L. U. Buchanan C. L. Duncan A. R. (2010). *ANTIBODIES TO TGF-β. US Patent, US7723486B2.*

[B27] Liu R. M. Desai L. P. (2015). Reciprocal regulation of TGF-β and reactive oxygen species: a perverse cycle for fibrosis. *Redox Biol.* 6 565–577. 10.1016/j.redox.2015.09.009 26496488 PMC4625010

[B28] Lokau J. Garbers C. (2020). Biological functions and therapeutic opportunities of soluble cytokine receptors. *Cytokine Growth Factor Rev.* 55 94–108. 10.1016/j.cytogfr.2020.04.003 32386776

[B29] López-Casillas F. Wrana J. L. Massagué J. (1993). Betaglycan presents ligand to the TGF beta signaling receptor. *Cell* 73 1435–1444. 10.1016/0092-8674(93)90368-z 8391934

[B30] Malhi H. Gores G. J. (2008). Cellular and molecular mechanisms of liver injury. *Gastroenterology* 134 1641–1654. 10.1053/j.gastro.2008.03.002 18471544 PMC2553363

[B31] Mendoza V. Vilchis-Landeros M. M. Mendoza-Hernández G. Huang T. Villarreal M. M. Hinck A. P. (2009). Betaglycan has two independent domains required for high affinity TGF-beta binding: proteolytic cleavage separates the domains and inactivates the neutralizing activity of the soluble receptor. *Biochemistry* 48 11755–11765. 10.1021/bi901528w 19842711 PMC2796082

[B32] Milani S. Herbst H. Schuppan D. Stein H. Surrenti C. (1991). Transforming growth factors beta 1 and beta 2 are differentially expressed in fibrotic liver disease. *Am. J. Pathol.* 139 1221–1229. 1750499 PMC1886459

[B33] Moses H. L. Roberts A. B. Derynck R. (2016). The discovery and early days of TGF-β: a historical perspective. *Cold Spring Harb. Perspect. Biol.* 8:a021865. 10.1101/cshperspect.a021865 27328871 PMC4930926

[B34] Noyan F. Díez I. A. Hapke M. Klein C. Dewey R. A. (2012). Induced transgene expression for the treatment of solid tumors by hematopoietic stem cell-based gene therapy. *Cancer Gene Ther.* 19 352–357. 10.1038/cgt.2012.8 22402626

[B35] Povero D. Busletta C. Novo E. di Bonzo L. V. Cannito S. Paternostro C. (2010). Liver fibrosis: a dynamic and potentially reversible process. *Histol. Histopathol.* 25 1075–1091. 20552556 10.14670/HH-25.1075

[B36] Proetzel G. Pawlowski S. A. Wiles M. V. Yin M. Boivin G. P. Howles P. N. (1995). Transforming growth factor-beta 3 is required for second-ary palate fusion. *Nat. Genet.* 11 409–414. 7493021 10.1038/ng1295-409 PMC3855390

[B37] Rodríguez T. M. Saldías A. Irigo M. Zamora J. V. Perone M. J. Dewey R. A. (2015). Effect of TGF-β1 stimulation on the secretome of human adipose-derived mesenchymal stromal cells. *Stem Cells Transl. Med.* 4 894–898. 10.5966/sctm.2015-0012 26025982 PMC4511148

[B38] Roopenian D. C. Akilesh S. (2007). FcRn: the neonatal Fc receptor comes of age. *Nat. Rev. Immunol.* 7 715–725. 10.1038/nri2155 17703228

[B39] Rotzer D. Roth M. Lutz M. Lindemann D. Sebald W. Knaus P. (2001). Type III TGF-beta receptor-independent signalling of TGF-beta2 via TbetaRII-B, an alternatively spliced TGF-beta type II receptor. *EMBO J.* 20 480–490. 10.1093/emboj/20.3.480 11157754 PMC133482

[B40] Sancho P. Mainez J. Crosas-Molist E. Roncero C. Fernández-Rodriguez C. M. Pinedo F. (2012). NADPH oxidase NOX4 mediates stellate cell activation and hepatocyte cell death during liver fibrosis development. *PLoS One* 7:e45285. 10.1371/journal.pone.0045285 23049784 PMC3458844

[B41] Sanford L. P. Ormsby I. Gittenberger-de Groot A. C. Sariola H. Friedman R. Boivin G. P. (1997). TGFbeta2 knockout mice have multiple developmental defects that are non- overlapping with other TGFbeta knock- out pheno-types. *Development* 124 2659–2670. 10.1242/dev.124.13.2659 9217007 PMC3850286

[B42] Serini G. Gabbiana G. (1996). Modulation of alpha-smooth muscle actin expres-sion in fibroblasts by transforming growth factor-beta isoforms: an *in vivo* and *in vitro* study. *Wound Repair Regen.* 4 278–287. 10.1046/j.1524-475x.1996.40217.x 17177825

[B43] Shull M. M. Ormsby I. Kier A. B. Pawlowski S. Diebold R. J. Yin M. (1992). Targeted disruption of the mouse transforming growth factor- beta-1 gene in multifocal inflammatory disease. *Nature* 359 693–699. 10.1038/359693a0 1436033 PMC3889166

[B44] Takahashi K. Akatsu Y. Podyma-Inoue K. A. Matsumoto T. Takahashi H. Yoshimatsu Y. (2020). Targeting all transforming growth factor-β isoforms with an Fc chimeric receptor impairs tumor growth and angiogenesis of oral squamous cell cancer. *J. Biol. Chem.* 295 12559–12572. 10.1074/jbc.ra120.012492 32631954 PMC7476713

[B45] Tandale J. B. Badgujar S. B. Tandale B. U. Angre U. Daftary S. B. Lala S. (2020). An improved protocol for large scale production of high purity ‘Fc’ fragment of human immunoglobulin G (IgG-Fc). *J. Chromatogr. B Analyt. Technol. Biomed. Life Sci.* 1159:122400. 10.1016/j.jchromb.2020.122400 33126073

[B46] Verrecchia F. Mauviel A. (2007). Transforming growth factor-beta and fibro-sis. *World J. Gastroenterol.* 13 3056–3062. 17589920 10.3748/wjg.v13.i22.3056 PMC4172611

[B47] Walton K. L. Johnson K. E. Harrison C. A. (2017). Targeting TGF-β mediated SMAD signaling for the prevention of fibrosis. *Front. Pharmacol.* 8:461. 10.3389/fphar.2017.00461 28769795 PMC5509761

[B48] Wieser R. Wrana J. L. Massagué J. (1995). GS domain mutations that constitu-tively activate T beta R-I, the downstream signaling component in the TGF-beta receptor complex. *EMBO J.* 14 2199–2208. 10.1002/j.1460-2075.1995.tb07214.x 7774578 PMC398326

[B49] Wordinger R. J. Sharma T. Clark A. F. (2014). The role of TGF-β2 and bone morphogenetic proteins in the trabecular meshwork and glaucoma. *J. Ocul. Pharmacol. Ther.* 30 154–162. 10.1089/jop.2013.0220 24517218 PMC3991963

[B50] Yung L. M. Nikolic I. Paskin-Flerlage S. D. Pearsall R. S. Kumar R. Yu P. B. (2016). A selective transforming growth factor-β ligand trap atten-uates pulmonary hypertension. *Am. J. Respir. Crit. Care Med.* 194 1140–1151. 10.1164/rccm.201510-1955oc 27115515 PMC5114445

[B51] Zhang Y. E. (2017). Non-Smad signaling pathways of the TGF-b family. *Cold Spring Harb. Perspect. Biol.* 9:a022129. 10.1101/cshperspect.a022129 27864313 PMC5287080

[B52] Zhou X. Wang J. L. Lu J. Song Y. Kwak K. S. Jiao Q. (2010). Reversal of cancer cachexia and muscle wasting by ActRIIB antagonism leads to prolonged survival. *Cell* 142 531–543. 10.1016/j.cell.2010.07.011 20723755

