# Supplementary material for: A Novel Splice Variant of Human TGF-β Type II Receptor Encodes a Soluble Protein and Its Fc-Tagged Version Prevents Liver Fibrosis in vivo
Source: Front Cell Dev Biol. 2021 Sep 10;9:690397. doi: 10.3389/fcell.2021.690397 (PMC8461249; doi:10.3389/fcell.2021.690397)
Supplement: Supplementary file 1 [file Data_Sheet_1.pdf]

Supplementary Materials for

A novel splice variant of human TGF-β type II receptor encodes a soluble protein and its Fc-tagged version prevents liver fibrosis in vivo.

Marcela S. Bertolio, Anabela La Colla, Alejandra Carrea, Ana Romo, Gabriela Canziani, Stella M. Echarte, Sabrina Campisano, German P. Barletta, Alexander M. Monzon, Tania M. Rodríguez, Andrea N. Chisari, Ricardo A. Dewey.
Correspondence to: ricardodewey@intech.gov.ar

This PDF file includes:

Materials and methods
Figure. S1
Figure. S2
Figure. S3
Figure. S4
Table. S1
Table. S2
Supplementary Text

Materials and methods

Molecular dynamic simulation

In order to evaluate our TβRII-SE 3D model under physiological conditions, we performed a molecular dynamic (MD) simulation on the best model using the AMBER 16 software package.[1,2] Ions were added for charge neutralization. Each system was solvated with explicit TIP3P[3] water molecules in a truncated octahedral periodic box large enough to contain the protein and 10 Å of solvent on all sides. The all-hydrogen topology was obtained with the Amber ff14SB[4,5] force field. A 2 fs time step was used and the SHAKE algorithm was applied to all bonds involving hydrogen. Periodic boundary conditions and particle-mesh Ewald (PME) sums were used and a cut-off of 10 Å was applied to non-bonded interactions.

Minimization was performed by 100-step of steepest-descent and 400-step conjugate gradient minimizations applying constraints to the protein atoms. This was followed by a 400-step unconstrained conjugate gradient minimization.

The system was then heated for 150 ps until it reached the final temperature of 300 K. During heating, a harmonic restraint of 50.0 kcal/(mol·Å2) was applied to the protein atoms.

The system was equilibrated at constant pressure using 29 steps of 100 ps and reducing the restraint on each step. After the last step with restraints, all restraints were lifted and a final equilibration run was performed until reaching 5 ns of total equilibration time at a constant temperature of 300 K using an Andersen barostat and a Langevin thermostat with a γ collision frequency of 2 ps−1. Finally, a 1000 ns MD simulation is performed, collecting equilibrated configurations at 10 ps intervals.

Figure. S1.

```
TβRII-SE   ACCGGT------ATGGGTCGGGGGCTGCTCAGGGGCCTGTGGCCGCTGCACATCGTCCTGTGGACGCGTATCGCCAGCAC
coTβRII-SE ACCGGTGCCACCATGGGAAGAGGTCTCCTCAGAGGACTCTGGCCACTGCACATCGTCCTGTGGACCAGAATCGCATCTAC

TβRII-SE   GATCCCACCGCACGTTCAGAAGTCGGTTAATAACGACATGATAGTCACTGACAACAACGGTGCAGTCAAGTTTCCACAAC
coTβRII-SE CATCCCTCCTCATGTGCAGAAATCTGTCAACAATGACATGATCGTCACAGACAACAACGGTGCTGTGAAGTTTCCTCAGC

TβRII-SE   TGTGTAAATTTTGTGATGTGAGATTTTCCACCTGTGACAACCAGAAATCCTGCTTCTCCAAAGTGCATTATGAAGGAAAA
coTβRII-SE TGTGTAAGTTCTGCGACGTCAGGTTCAGTACCTGCGACAATCAGAAATCTTGTTTCAGCAAGGTGCACTACGAAGGGAAG

TβRII-SE   AAAAAAGCCTGGTGA
coTβRII-SE AAGAAAGCATGGAGATCT
```

TβRII-SE codon optimization. cDNA alignment showing changes made to TβRII-SE (yellow shading). To get coTβRII-SE, a Kozak consensus sequence (red nucleotides) was included. Additionally, some nucleotides have been substituted to make translation more efficient in human cells. To allow fusion in frame of cDNA with the human IgG-Fc domain cDNA, the stop codon of TβRII-SE was removed (italics) and replaced by a *Bgl*II recognition sequence in the new construct.

Figure. S2.

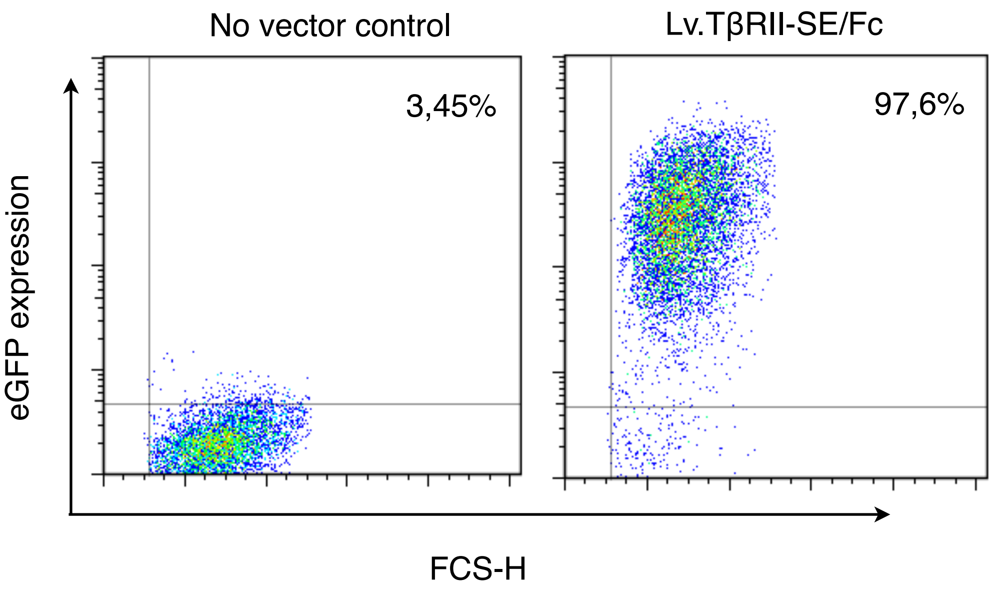

Transduction efficiency determination of Lv.TβRII-SE/Fc used to purify recombinant fusion proteins. Representative flow cytometry dot plots of 293T cells untransduced (no vector control) and transduced with Lv.TβRII-SE/Fc.

Figure. S3.

Mock **Lv.TβRII-SE/Fc**

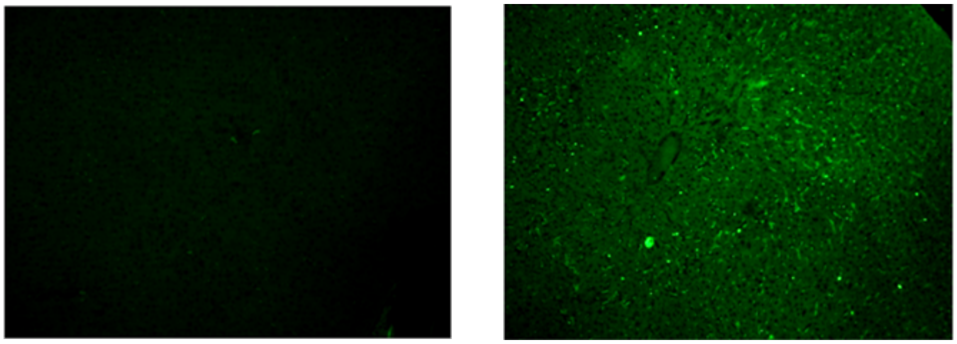

***In vivo* TβRII-SE/Fc liver transduction efficiency in rats.** Liver sections from normal and Lv.TβRII-SE/Fc-injected rats were employed to visualize eGFP-derived green fluorescence using fluorescence microscopy.

Figure. S4.

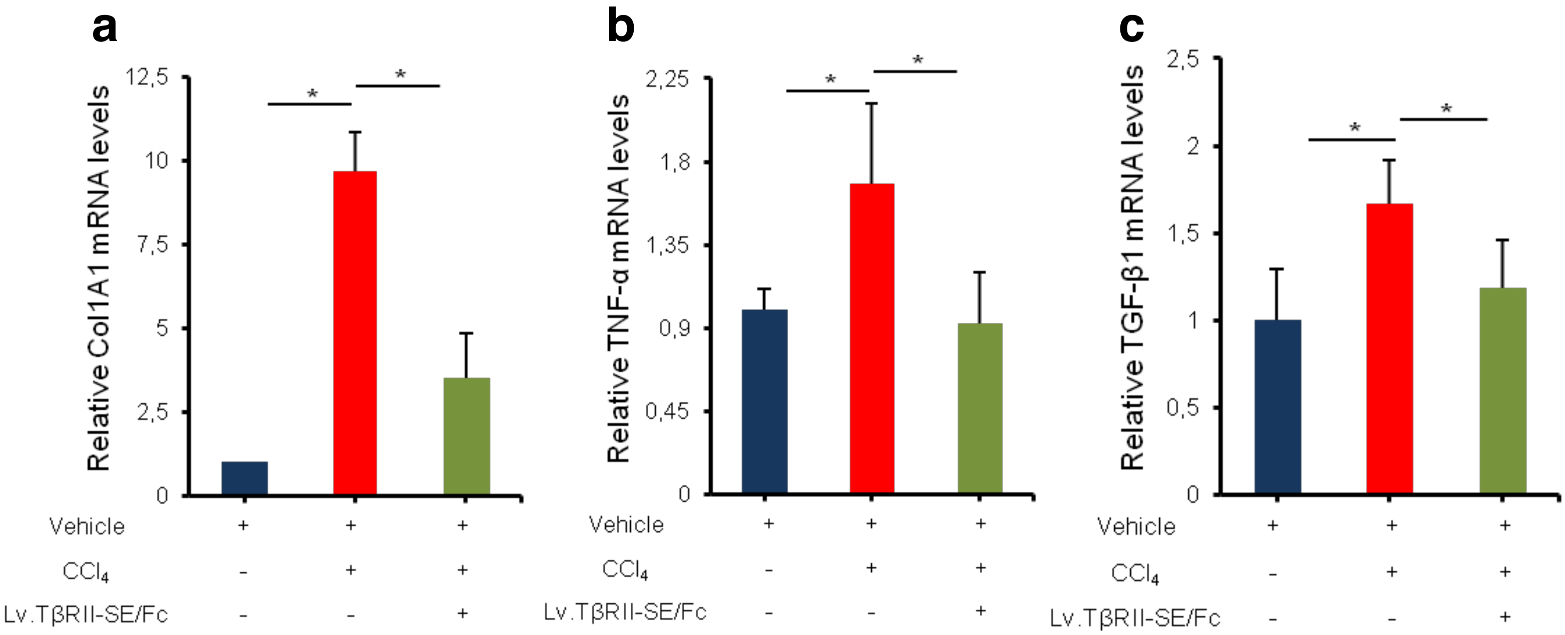

RT-qPCR determinations in rat liver cells from groups Vehicle, CCL_4 only, and CCL_4+ Lv.TβRII-SE/Fc. a Col1A1 (A), **b** TNF-α, and **c** TGF-β1. β-actin was used as a reference gene. The comparative CT method was employed for relative quantification. *$p<0.05$

**pI/Mw**	9.64/9161.72 Da	
**Signal peptide clivage site**	Between T23-I24	SignalP 4.1 Server [6] http://www.cbs.dtu.dk/services/SignalP/
**pI/Mw without signal peptide**	9.05/6532.51 Da	
**Glycation**	K46, 52, 78	NetGlycate 1.0 Server [7] http://www.cbs.dtu.dk/services/NetGlycate/
**Kinase-specific Phosphorylation**	S22, 31, 59, 66, 69 T18, 23, 39, 60, Y73	GPS web server 5.0 [8] http://gps.biocuckoo.org/online.php
**Sumoylation**	K76, 77, 78 (Non consensus)	GPS-SUMO 2.0 Online Service [9] http://sumosp.biocuckoo.org/online.php
**C-mannosylation**	No sites	NetCGlyc 1.0 Server [10] http://www.cbs.dtu.dk/services/NetCGlyc/
**N-Glycosylation**	No sites	NetNGlyc 1.0 Server http://www.cbs.dtu.dk/services/NetNGlyc/
**GalNAc O-glycosylation**	No sites	NetOGlyc 4.0 Server [11] http://www.cbs.dtu.dk/services/NetOGlyc/
**N-terminal Myristoylation**	No sites	Myristoylator [12] https://web.expasy.org/cgi-bin/myristoylator/myristoylator.pl
**Palmitoylation**	No sites	CSS-Palm 2.0 [13] http://csspalm.biocuckoo.org/online.php

Table. S1.

TβRII-SE predicted characteristics, including post-translational modifications.

Table. S2.

Ligands	Surface Density (RU/Kda)	Theoretical R_{max}	Experimental R_{max}	Stoichiometry experimental/ predicted
**TβRII-SE/Fc**	2.0	60.0	65.7 ± 12.9	1.1/1
	2.5	62.5	59.6 ± 9.3	0.95/1
	3.0	90.0	59.2 ± 7.3	0.95/1
**1D11**	0.8	80.0	126 ± 4.5	2.5/2
	1.0	100.0	120 ± 6.0	2.4/2
	2.4	120.0	60.8 ± 9.9	1.0/2
**TβRII-Fc**	0.9	22.5	44.5± 1.9	2.0/1
	1.0	25.0	48.2 ± 1	1.9/1
	1.2	30.0	57.2 ± 2.3	1.9/1

Stoichiometries of binding of TGF-β dimers to their experimental ligands.

Supplementary Text

References

1. Salomon-Ferrer, R., Case, D., & Walker, R. An Overview of the Amber Biomolecular Simulation Package. *WIREs Comput. Mol. Sci.* **3,** 198–210 (2013).
2. Case, D. *et al.* The Amber Biomolecular Simulation Programs. *J. Comput. Chem.* **26,** 1668–1688 (2005).
3. Jorgensen, W., Chandrasekhar, J., Madura, J., Impey, R., & Klein, M. Comparison of Simple Potential Functions for Simulating Liquid Water. *J. Chem. Phys.* **79,** 926–935 (1983).
4. Ponder, J., & Case, D. Force Fields for Protein Simulations. *Adv. Protein Chem.* **66,** 27–85 (2003).
5. Maier, J., *et al.* ff14SB: Improving the Accuracy of Protein Side Chain and Backbone Parameters from ff99SB. *J. Chem. Theory Comput.* **11,** 3696–3713 (2015).
6. Petersen, T. N., Brunak, S., von Heijne, G., & Nielsen, H. SignalP 4.0: discriminating signal peptides from transmembrane regions. *Nat. Methods* **8,** 785-786 (2011).
7. Johansen, M. B., Kiemer, L., & Brunak, S. Analysis and prediction of mammalian protein glycation. *Glycobiology* **16,** 844-853 (2006).
8. Wang, C., *et al.* GPS 5.0: An Update on the Prediction of Kinase-specific Phosphorylation Sites in Proteins. *Genomics Proteomics Bioinformatics* ***18,*** 72–80 (2020).
9. Zhao, Q., *et al.* GPS-SUMO: a tool for the prediction of sumoylation sites and SUMO-interaction motifs. *Nucleic Acids Res.* **42** (Web Server issue), W325–W330 (2014).
10. Julenius K. NetCGlyc 1.0: prediction of mammalian C-mannosylation sites. *Glycobiology* **17,** 868–876 (2007).
11. Pedersen, N. B., *et al.* Precision mapping of the human O-GalNAc glycoproteome through Simple Cell technology. *EMBO J.* **32,** 1478–1488 (2013).
12. Bologna, G., Yvon, C., Duvaud, S., & Veuthey, A. L. N-Terminal myristoylation predictions by ensembles of neural networks. *Proteomics* **4,** 1626–1632 (2004).
13. Ren, J., *et al.* (2008). CSS-Palm 2.0: an updated software for palmitoylation sites prediction. *Protein Eng Des Sel.* **21,** 639–644 (2008).
